# Fault Ride-Through Optimization Scheme for Hybrid AC/DC Transmission Systems on the Same Tower

**DOI:** 10.3390/s25196216

**Published:** 2025-10-07

**Authors:** Xu Chu, Qi Liu, Letian Fu, Shaoshuai Yu, Weidong Wang

**Affiliations:** The College of Electrical and Information Engineering, Hunan University, Changsha 410082, China; xu.chu@hnu.edu.cn (X.C.); fuletian@hnu.edu.cn (L.F.); yshaoshuai@foxmail.com (S.Y.); dwwang@hnu.edu.cn (W.W.)

**Keywords:** LCC-HVDC, FRT, power compensation, protection-control cooperation

## Abstract

Sensors in power systems utilize the detection results of fault signals to guide subsequent fault handling procedures. However, the traditional phase-shift restart strategy exhibits limitations such as power interruptions, reactive power redundancy, and intersystem fault clearance failures when addressing faults in the hybrid AC/DC transmission system. To address these shortcomings, a power compensation-based fault ride-through (FRT) scheme and a protection-control cooperation FRT scheme are proposed, taking into account the operational characteristics of the symmetric monopole LCC-HVDC (SM-LCC-HVDC). The power compensation-based FRT scheme actively compensates for power, mitigating the impact of reactive power redundancy on the AC-side bus during faults. The protection-control cooperation FRT scheme is activated after sufficient AC components are detected on the DC side. It leverages the adjustability of the DC system to proactively activate protection for AC transmission lines. An electromagnetic transient simulation model of the hybrid AC/DC same-tower transmission system was developed in PSCAD/EMTDC. Simulation results validate the effectiveness and superiority of the proposed methods.

## 1. Introduction

HVDC transmission technology has been widely employed in cross-regional power delivery [1], interconnection of large power grids [2], integration of distributed energy resources [3], island power supply [4], and megacity electrification [5], owing to its significant advantages including low line losses, long transmission distance, large transmission capacity, non-synchronous operation, and rapid power regulation. Overhead DC transmission lines, exposed to the atmosphere, operate under harsh conditions influenced by regional climate and environmental factors, making them the component with the highest failure probability in the entire transmission system [6]. DC line faults can jeopardize the safe operation of electrical equipment, easily induce commutation failures, interrupt normal power transmission, and lead to DC system blockades or shutdowns. These events may trigger system voltage and frequency stability issues, potentially resulting in widespread blackouts [7]. Therefore, the fault ride-through and restart capability of DC transmission lines is critically important for ensuring equipment safety, enhancing power supply reliability, and maintaining overall system stability [8].

Conventional Line Commutated Converter based High Voltage Direct Current (LCC-HVDC) systems typically rely on the DC-line fault recovery sequences (DLFRS) of the pole control system to clear and recover from DC line faults. This process primarily consists of three stages: phase-shifting, de-ionization, and restart [9]. Decisions on whether to initiate a restart, the setting of the de-ionization time, and the number of restart attempts must be determined based on actual operating conditions. Currently, the probability of transient faults on DC transmission lines is as high as 90%. For the vast majority of faults, executing the DLFRS once can quickly clear the fault and restore power supply. Systems are generally configured to allow multiple DLFRS attempts until a successful restart is achieved; however, if restart failures persist after reaching the maximum allowable number of attempts, the fault is deemed permanent, prompting the control and protection system to initiate a blockade sequence and shut down the DC system.

Currently, most HVDC projects predominantly adopt a symmetrical bipolar topology. Existing literature has primarily focused on fault clearance and restart strategies for symmetrical bipolar HVDC systems. Reference [10] designed two distinct fault restart logics specifically for DC line transient faults and DC valve group faults, respectively; results demonstrated that this approach contributes to enhancing the system availability and operational performance of the DC system. Reference [11] introduced the phase-shift fault clearance strategy from LCC-HVDC into hybrid HVDC systems, coordinating with the circuit breaker on the modular multilevel converter (MMC) side to achieve fault clearance. Reference [12] analyzed the gradient characteristics of the DC voltage on the inverter side (I-side) during LCC-HVDC DC line faults, established an identification criterion for distinguishing between transient and permanent faults, and enabled the selective activation of restart strategies, thereby improving their reliability. Reference [13] utilized the healthy-pole converter to inject DC voltage disturbances into the line, inducing characteristic signals within the faulty line. Information regarding the fault nature was extracted from these signals to achieve selective restart of the DC line. Reference [14] proposed a control mode switching method for bipolar HVDC systems, enabling the faulty-pole converter to operate as a STATCOM to support the AC grid. This enhances the active power transmission capability of the healthy-pole converter, thereby mitigating the impact of power imbalance between the affected offshore wind farms and the converter station.

In recent years, to accommodate the integration of large-scale renewable energy into the power grid, enhancing the transmission capacity of critical sections and improving the flexibility and controllability of the grid have become urgent issues to address [15,16]. The scheme of converting existing AC transmission lines into SM-LCC-HVDC lines to significantly increase transmission capacity within limited corridor space has attracted active attention and exploration from researchers and experts [17,18,19,20]. Given the relatively short transmission distance and modest capacity requirements of the project, the DC transmission line is planned to adopt a symmetrical monopolar topology and be co-located on the same tower with AC lines in a hybrid AC/DC shared-tower configuration [21]. In SM-LCC-HVDC systems, the neutral points at the two ends lack a direct connection, leading to different fault characteristics under single-pole DC faults compared to symmetrical bipolar LCC-HVDC systems. Consequently, the employed DC fault clearance strategies require adjustments. Furthermore, AC/DC intersystem faults (ISFs), as a distinctive fault type unique to hybrid shared-tower transmission systems, involve the coordinated response of two different power source systems for their clearance. Reference [22] proposed a coordinated protection scheme between AC and DC systems for AC-DC ISFs; however, it did not explore the adaptability of the DLFRS under such fault scenarios.

References [23,24] proposed novel FRT schemes for Offshore Wind Farms and DFIG-Based Wind Turbines, respectively. Both approaches are implemented via sensors, paving new avenues for the optimization of sensor applications. Accordingly, this paper first analyzes the characteristics of DC single-pole faults in SM-LCC-HVDC systems and discusses the applicability of conventional phase-shift strategies to such systems. To address the issue of complete power loss in SM-LCC-HVDC during phase-shift, a novel power compensation-based FRT strategy is proposed. Subsequently, the FRT requirements of hybrid AC/DC systems under ISF scenarios are clarified. Then, aiming to achieve rapid and reliable fault clearance after AC/DC ISFs, a protection-control cooperation FRT strategy is developed. Finally, simulations are conducted to verify the effectiveness and superiority of the proposed method.

## 2. Limitations of the Phase-Shift FRT in SM-LCC-HVDC Systems and Corresponding Improvements

### 2.1. Fault Types of Hybrid AC/DC Transmission Systems

**Figure 1:**
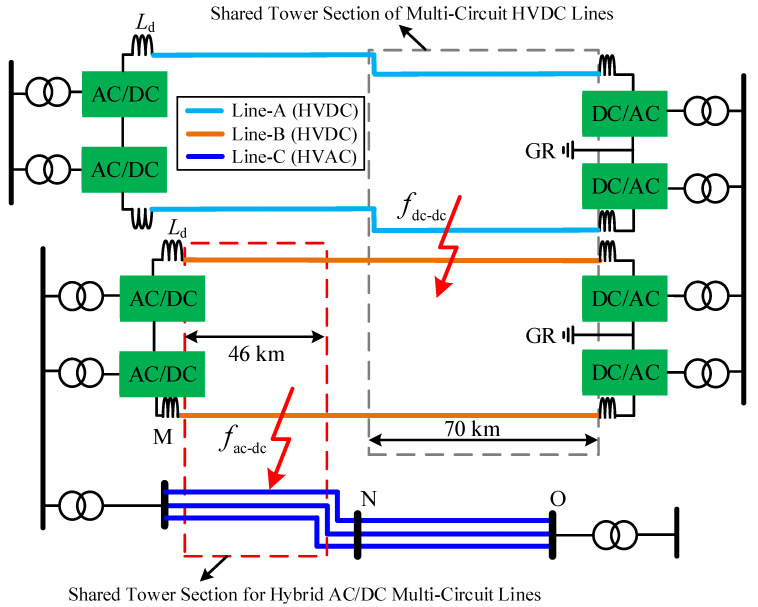
Hybrid AC/DC transmission systems on the same tower.

A section of the multi-circuit AC transmission lines within the same corridor has been converted into a SM-LCC-HVDC system as illustrated in Figure 1. The DC transmission lines are embedded within the AC grid, forming the hybrid AC/DC multi-circuit transmission system. In densely populated transmission line corridors, to conserve land use and reduce project investment, the SM-LCC-HVDC system is configured with a grounding electrode exclusively at the inverter side. Under this configuration, electrical coupling exists between the DC bipolar poles at the rectifier side, resulting in fault characteristics of the SM-LCC-HVDC system that diverge from those of conventional bipolar HVDC systems. These differences will be discussed in detail in the following section. Here, Line-A and Line-B represent two HVDC overhead lines, while Line-C denotes an HVAC overhead line, the shared tower sections among them are marked with dashed boxes. The HVAC system is a three-phase transmission line with its neutral point directly grounded. The parameters of the system shown in Figure 1 are provided in Table 1, and the modeling of this system has been completed in the PSCAD/EMTDC simulation software version 4.5.

Line faults within a hybrid AC/DC overhead transmission system include AC line faults, DC line faults, DC-DC ISFs, and AC-DC ISFs. With the exception of AC line faults, all other fault types will trigger the FRT mechanism in the DC system. Subsequently, the fault current is cleared. The uniqueness of a DC-DC ISF (indicated as $f_{\mathrm{dc}-\mathrm{dc}}$ in Figure 1) lies in the fact that both DC systems affected by the fault need to execute their respective FRT procedures. In contrast, an AC-DC ISF (indicated as $f_{\mathrm{ac}-\mathrm{dc}}$ in Figure 1) not only requires the DC system to perform FRT but also involves coordination with AC-side protection schemes. It is noteworthy that in fault scenarios involving multi-phase touching between DC and AC lines, the condition is equivalent to a phase-to-phase short circuit on the AC side. Consequently, AC protection schemes will respond rapidly to trip the AC circuit breakers, thereby disconnecting the AC power sources from the fault loop. Conversely, when both poles of a DC line touching an AC line simultaneously, it constitutes a pole-to-pole short circuit on the DC side. This event triggers the rapid blocking of both DC poles, disconnecting the DC power sources from the fault path. Therefore, among all types of AC-DC ISFs, the challenge of difficult fault clearance is primarily concentrated in the single-pole-to-single-phase fault scenario. Hence, this paper focuses specifically on this single-pole-to-single-phase type of AC-DC inter-system fault.

### 2.2. Adaptability Analysis of Phase-Shifting FRT Applied to SM-LCC-HVDC Systems

**Figure 2:**
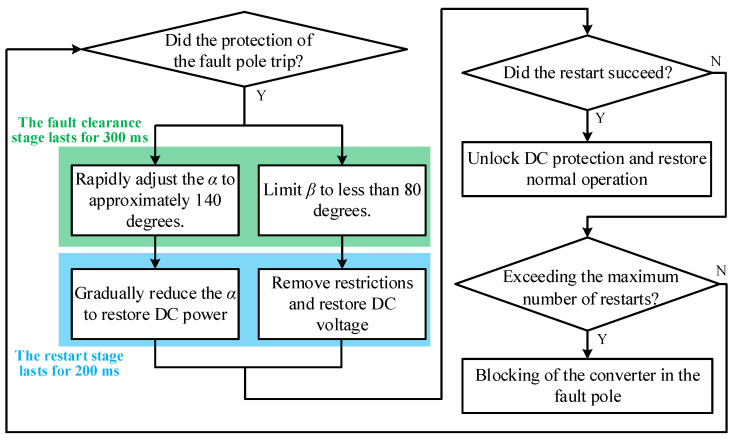
Flowchart of conventional phase-shifting FRT.

The phase-shift restart strategy is widely employed for line fault clearing and restart in conventional bipolar LCC-HVDC systems, with its workflow illustrated in Figure 2. Upon fault detection, the rectifier of the fault pole immediately increases its firing angle command to approximately 140 degrees to briefly enter an inverter operation mode. At this point, its DC-side voltage becomes negative. Simultaneously, the inverter is assigned a maximum limit of around 80 degrees to prevent the inverter voltage from reversing. Under the combined action of the reverse voltage from the rectifier and the voltage from the inverter, the energy stored in the fault line is fed back into the AC system through both converters, leading to a rapid decline in the fault current. Additionally, the unidirectional conduction characteristic of thyristors ensures that the fault current cannot reverse, thereby enabling rapid clearance of the DC fault.

**Figure 3:**
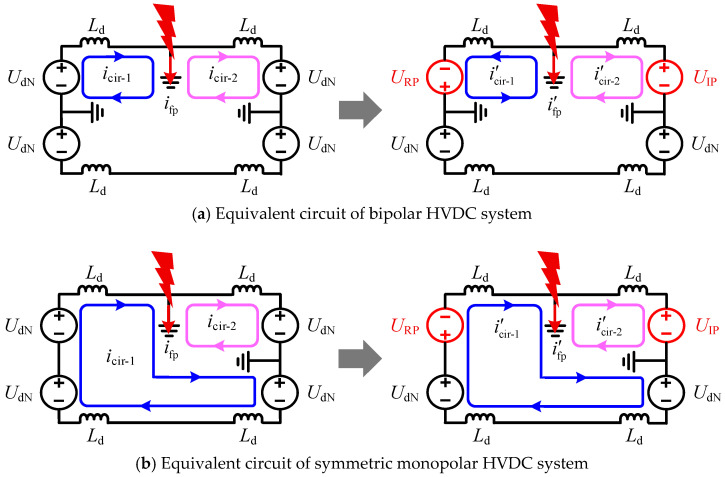
Equivalent circuits of the HVDC systems under the conventional phase-shifting strategy.

However, significant differences exist in the transient characteristics of line faults between symmetric monopolar and bipolar DC systems. Under single-pole fault scenarios, the equivalent circuits of both systems before and after phase-shifting are illustrated in Figure 3. As shown, the fault point serves as a boundary that delineates two distinct fault current loops in both systems during single-pole fault conditions.

During the initial stage of the fault, prior to the intervention of system controls, the DC-side voltage remains essentially at its rated value $U_{\mathrm{dN}}$. Neglecting the DC filter and the equivalent line inductance, the expression for the fault loop current before phase-shifting is given by:

(1) $$i_{\mathrm{cir}-1}\approx I_{\mathrm{dN}}+1/3L_{d}\int_{t_{0}}^{t_{0}+\Delta t}U_{\mathrm{dN}}dt$$

(2) $$i_{\mathrm{cir}-2}\approx I_{\mathrm{dN}}-\frac{1}{L_{d}}\int_{t_{0}}^{t_{0}+\Delta t}U_{\mathrm{dN}}dt$$

Here, $i_{\mathrm{cir}-1}$ and $i_{\mathrm{cir}-2}$ respectively represent the currents of the two current loops, $I_{\mathrm{dN}}$ represents the rated current for normal operation, $t_{0}$ represents the current during a fault, and Δt represents the sampling interval of the system.

Following the fault, $i_{\mathrm{cir}-1}$ exhibits an upward trend while $i_{\mathrm{cir}-2}$ shows a downward trend, with the rate of change of $i_{\mathrm{cir}-2}$ being approximately three times that of $i_{\mathrm{cir}-1}$. The expression for the fault loop current after phase-shifting is given by:

(3) $${i^{\prime }}_{\mathrm{cir}-1}\approx I_{\mathrm{dN}}-\frac{1}{3L_{d}}\int_{t_{0}}^{t_{0}+\Delta t}U_{\mathrm{RP}}dt$$

(4) $${i^{\prime }}_{\mathrm{cir}-2}\approx I_{\mathrm{dN}}-\frac{1}{L_{d}}\int_{t_{0}}^{t_{0}+\Delta t}U_{\mathrm{IP}}dt$$

Based on Equations (1) to (4), the expressions for the fault point currents $i_{\mathrm{fp}}$ and ${i^{\prime }}_{\mathrm{fp}}$ before and after phase-shifting can be derived as follows:

(5) $$i_{\mathrm{fp}}=i_{\mathrm{cir}-1}-i_{\mathrm{cir}-2}\approx \frac{4}{3L_{d}}\int_{t_{0}}^{t_{0}+\Delta t}U_{\mathrm{dN}}dt$$

(6) $${i^{\prime }}_{\mathrm{fp}}={i^{\prime }}_{\mathrm{cir}-1}-{i^{\prime }}_{\mathrm{cir}-2}\approx \frac{1}{L_{d}}\int_{t_{0}}^{t_{0}+\Delta t}U_{\mathrm{IP}}dt-\frac{1}{L_{d}}\int_{t_{0}}^{t_{0}+\Delta t}\frac{U_{\mathrm{RP}}}{3}dt$$

Since $U_{\mathrm{IP}}\gg U_{\mathrm{RP}}/3$, ${i^{\prime }}_{\mathrm{fp}}$ remains consistently greater than zero. Consequently, this finding indicates that if the conventional bipolar phase-shift strategy is applied to SM-LCC-HVDC systems, it cannot effectively eliminate the current at the fault point, proving ineffective for fault clearance.

**Figure 4:**
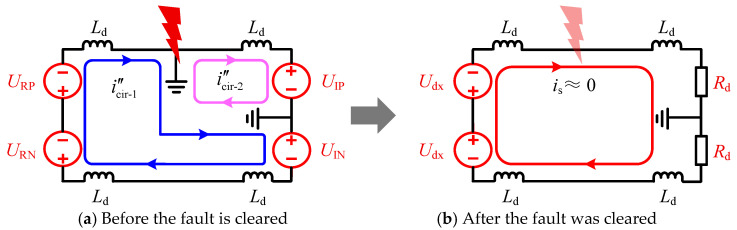
Phase-shifting strategy applicable to the SM-LCC-HVDC system.

If phase-shift operation is simultaneously applied to both converters at the rectifier side (R-side) in the SM-LCC-HVDC system, the equivalent circuit of the system is illustrated in Figure 4a. The current expressions for the ${i^{\prime \prime }}_{\mathrm{cir}-1}$ and the ${i^{\prime }}_{\mathrm{fp}}$ after phase-shifting are given as follows:

(7) $${i^{\prime \prime }}_{\mathrm{cir}-1}\approx I_{\mathrm{dN}}-\frac{1}{3L_{d}}\int_{t_{0}}^{t_{0}+\Delta t}(U_{\mathrm{RP}}+U_{\mathrm{RN}}+U_{\mathrm{IN}})dt$$

(8) $${i^{\prime }}_{\mathrm{fp}}={i^{\prime \prime }}_{\mathrm{cir}-1}-{i^{\prime \prime }}_{\mathrm{cir}-2}\approx \frac{1}{L_{d}}\int_{t_{0}}^{t_{0}+\Delta t}U_{\mathrm{IP}}dt-\frac{1}{L_{d}}\int_{t_{0}}^{t_{0}+\Delta t}(\frac{U_{\mathrm{RP}}+U_{\mathrm{RN}}+U_{\mathrm{IN}}}{3})dt$$

Under this phase-shift strategy, the fault point current can be suppressed provided that $U_{\mathrm{IP}}\approx (U_{\mathrm{RP}}+U_{\mathrm{RN}}+U_{\mathrm{IN}})/3$. The fault loop current exhibits a trend of gradually decreasing from its rated value to zero. As the line current approaches zero, the converters operate in the discontinuous conduction mode. The R-side outputs a low negative voltage, while the inverter-side converter can be equivalently represented as a sizeable resistance. If the fault is temporary, the arc will extinguish after the fault current is limited to zero, allowing the line to restore its insulation capability. The equivalent circuit of the system after fault clearance is illustrated in Figure 4b.

Based on the preceding analysis, it is established that during the phase-shift ride-through operation, the controlled reduction of the bipolar current to zero results in an interruption of the active power transmitted by the entire system. Consequently, the reactive power previously consumed by the converters becomes redundant. Neglecting fluctuations in AC grid voltage and firing angle (or extinction angle) during the transient process, the expression for the redundant reactive power at the converter station under rapid phase-shift control is derived as follows:

(9) $$Q_{\mathrm{RO}}\approx P_{\mathrm{dN}}\times \sqrt{\frac{U_{\mathrm{dRO}}^{2}}{U_{\mathrm{dN}}^{2}}-1}=P_{\mathrm{dN}}\times \sqrt{\frac{{\left(3\sqrt{2}/\pi \times K_{\mathrm{TR}}U_{\mathrm{acg}}\right)}^{2}}{U_{\mathrm{dN}}^{2}}-1}$$

(10) $$Q_{\mathrm{IO}}\approx P_{\mathrm{dN}}\times \sqrt{\frac{U_{\mathrm{dRO}}^{2}}{U_{\mathrm{dN}}^{2}}-1}=P_{\mathrm{dN}}\times \sqrt{\frac{{\left(3\sqrt{2}/\pi \times K_{\mathrm{TI}}U_{\mathrm{acg}}\right)}^{2}}{U_{\mathrm{dN}}^{2}}-1}$$

where $Q_{\mathrm{RO}}$ and $Q_{\mathrm{IO}}$ represent the redundant reactive power at the rectifier station and inverter station during the phase-shift ride-through operation, respectively; $U_{\mathrm{dRO}}$ and $U_{\mathrm{dIO}}$ denote the no-load DC voltages of the rectifier and inverter, respectively; $P_{\mathrm{dN}}$ is the rated active power; $U_{\mathrm{acg}}$ represents the AC-side voltage; $K_{\mathrm{TR}}$ and $K_{\mathrm{TI}}$ indicate the transformer ratios at the R-side and I-side, respectively.

**Figure 5:**
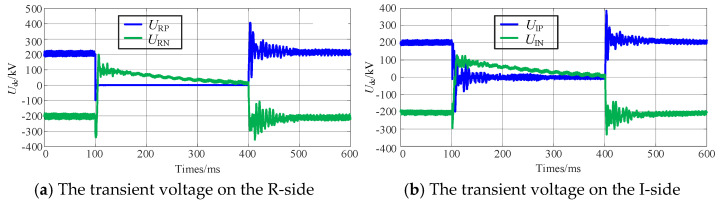

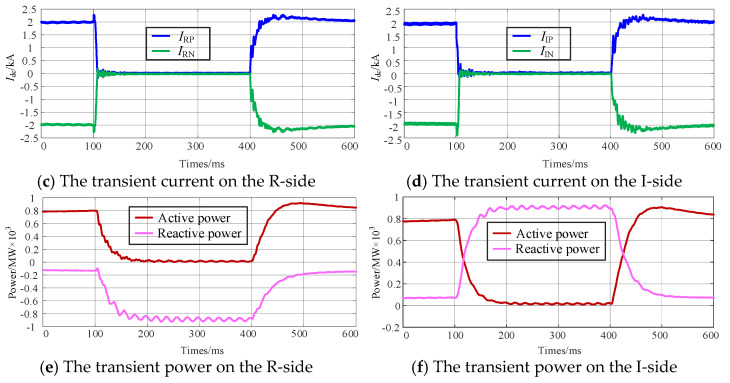
Transient electrical quantities during the SM−LCC−HVDC phase-shifting strategy.

Taking the temporary grounding fault on the positive pole of Line-B in Figure 1 as an example, the transient voltage, current, and power variations of Line-B during the FRT process are illustrated in Figure 5, with the fault inception time set at 100 ms.

As illustrated in Figure 5, the voltage of the fault-pole rapidly drops to zero under the influence of the fault point, while the phase-shifted voltage of the non-fault-pole promptly reverses its polarity and subsequently decays to zero under the effect of opposite-polarity sources at both terminals. Furthermore, the currents in both the faulty and non-fault-poles at each end decrease rapidly to zero, and the current at the fault point is effectively suppressed. During this process, the transmitted active power of the system drops to zero, while the reactive power increases to nine times its pre-fault value.

Although this strategy in SM-LCC-HVDC can suppress the electric arc at the fault point, it has significant drawbacks: during the phase-shifting process, the surplus reactive power can cause severe overvoltage in the AC system, which is particularly pronounced in weak AC systems. Moreover, the phase shift applied to the non-fault-pole will result in the healthy line being unable to transmit active power, thereby imposing a substantial active power impact on both the sending and receiving AC systems.

### 2.3. Power Compensation-Based FRT Strategy

**Figure 6:**
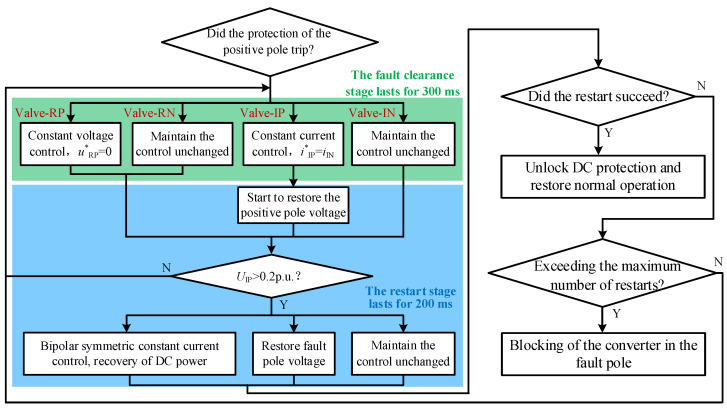
Flowchart of the power compensation-based FRT scheme for SM-LCC-HVDC.

To achieve uninterrupted power transfer during the DLFRS, mitigate the impact on the AC side, and ensure a discharge path for lightning strike energy, this section proposes to utilize the healthy pole line—which remains capable of power transmission—to fully compensate for the active power loss incurred during the FRT period. As shown in Figure 4, Valve-RP, Valve-RN, and Valve-IN can each regulate the $i_{\mathrm{cir}-1}$, while Valve-IP can regulate the current in $i_{\mathrm{cir}-2}$. Therefore, by controlling the two converters at the inverter side, equal current can be maintained in both loops, thereby achieving zero current at the fault point. The voltage of the negative pole line can be controlled either by the converter on the R-side or by the non-faulty pole converter on the N-side. The specific workflow of the power compensation-based ride-through scheme proposed in this section is illustrated in Figure 6. Here, a positive pole ground fault is taken as an example; for a negative pole ground fault, a symmetrical strategy can be adopted to achieve fault ride-through and restart.

**Figure 7:**
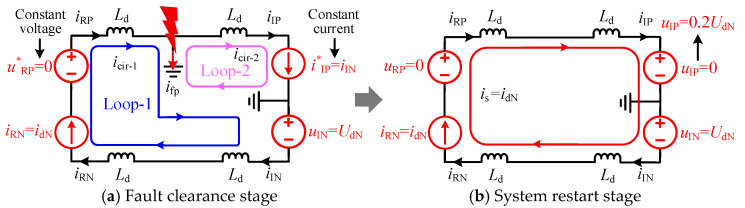
Fault equivalent circuit of the power compensation-based FRT scheme for SM-LCC-HVDC.

During the fault clearance phase, the non-faulty pole converter maintains its original control strategy. The converter of the faulty pole on the I-side is switched to constant current control, regulating its DC current to match that of the non-faulty pole. This ensures equal currents in both Loop-1 and Loop-2, thereby limiting the current at the fault point. The equivalent circuit corresponding to this operation is shown in Figure 7a. During the restart phase, the converter of the faulty pole on the I-side is switched to DC voltage control in an attempt to re-establish the line voltage, as depicted in Figure 7b. If the voltage established on the I-side exceeds 0.2 p.u., the R-side initiates the restoration of power and voltage. If the DC voltage of the faulty pole fails to be established, the converter on the I-side reverts to constant current control and returns to the fault clearance state.

**Figure 8:**
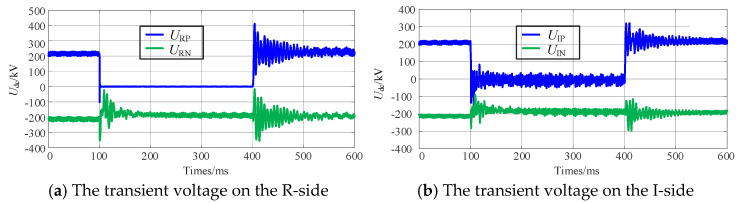

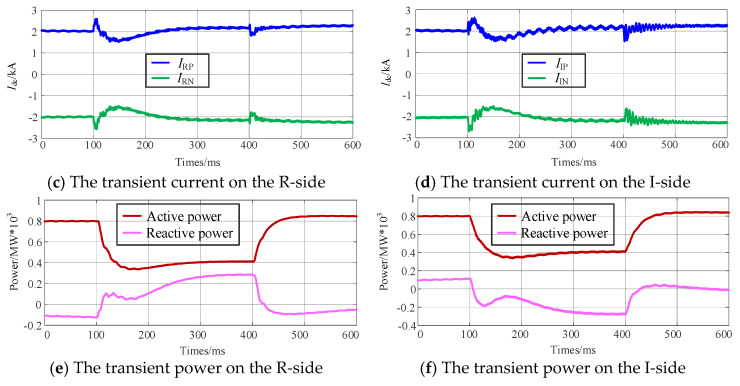
Transient electrical quantities during the SM−LCC−HVDC power compensation-based FRT.

An instantaneous ground fault is applied again at the positive pole of Line-B. The transient electrical quantity changes during the power compensation-based FRT are illustrated in Figure 8.

It can be observed that, after a brief oscillation following the initiation of the power compensation-based FRT strategy, both the voltage and current of the non-faulty pole remain stable at their rated values, while the currents output from the converters on both ends of the faulty pole remain equal. Throughout this process, the system is capable of transmitting 50% of the active power, and the resulting reactive power surplus is significantly smaller compared to the phase-shifting FRT.

## 3. Adaptive Analysis of FRT Strategy Under ISF

### 3.1. Core Requirements for FRT Under ISF

**Figure 9:**
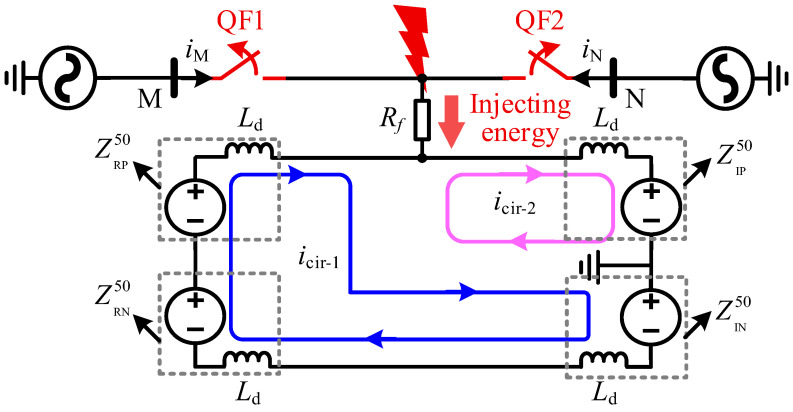
Equivalent circuit for fault clearance under AC-DC ISF.

In hybrid AC/DC overhead transmission lines, an inter-system fault can escalate from a single-system fault into a composite fault involving multiple systems, necessitating coordinated FRT efforts across these systems. In the context of a DC-DC ISF, the power compensation-based FRT strategy remains applicable. Both faulty DC systems can independently execute the procedure illustrated in Figure 6. For an AC-DC ISF, however, relying solely on the DC system for FRT is insufficient, especially since the electrical connection between the AC and DC systems remains intact. The continuous energy injection from the AC power source prevents the arc at the fault point from extinguishing, as illustrated in Figure 9, which ultimately impedes successful restart of the DC system.

Currently, dedicated methods for identifying AC-DC ISF generally require additional hardware investment or modifications to the existing logic of DC protection systems, and they have yet to undergo prolonged field operational validation. Therefore, in relatively conservative engineering contexts, there is a preference to maintain the existing AC and DC protection configurations and to rely on coordination between the AC and DC systems to clear ISFs. A crucial aspect of this approach is ensuring that AC line protection can rapidly and reliably trip AC circuit breakers in AC-DC ISFs.

### 3.2. Performance of Primary AC Protection Under AC-DC ISF

Longitudinal differential protection is typically employed as the primary protection for HVAC transmission lines. To ensure that the protection does not operate incorrectly during external faults, quantities $I_{d}=\left|{\dot{I}}_{M}+{\dot{I}}_{N}\right|$ and $I_{r}=\left|{\dot{I}}_{M}-{\dot{I}}_{N}\right|$ are designated as the differential and restraining quantities, respectively, resulting in a differential protection criterion with restraining characteristics as follows:

(11) $$\left\{\begin{array}{l} I_{d}>K_{br}I_{r}\\ I_{d}>I_{op} \end{array}\right.$$

where the value range of the braking coefficient $K_{br}$ is from 0.5 to 0.8, and $I_{op}$ is the threshold for the differential current to activate.

**Figure 10:**
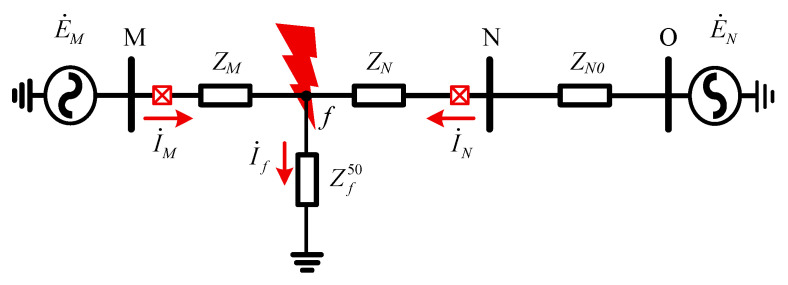
Power-frequency equivalent network of the system.

In practical engineering, protection devices typically employ the full-cycle Fourier algorithm to accurately extract the magnitude and phase of power-frequency components. $I_{d}$ and $I_{r}$ are then derived through phasor operations. The power-frequency equivalent impedance of the section between the AC-side fault point and the DC grounding point is denoted as $Z_{f}^{50}$, resulting in the power-frequency equivalent network of the system under fault conditions as illustrated in Figure 10.

Based on Figure 10, the steady-state differential quantity and restraining quantity for AC line MN were calculated as follows:

(12) $$I_{d}=\left|\frac{(Z_{N}+Z_{NO})\cdot {\dot{E}}_{M}+Z_{M}\cdot {\dot{E}}_{N}}{(Z_{M}+Z_{f})\cdot (Z_{N}+Z_{NO}+Z_{f})-Z_{f}^{2}}\right|$$

(13) $$I_{r}=\left|\frac{(Z_{N}+Z_{NO}+2Z_{f})\cdot {\dot{E}}_{M}-(Z_{M}+2Z_{f})\cdot {\dot{E}}_{N}}{(Z_{M}+Z_{f})\cdot (Z_{N}+Z_{NO}+Z_{f})-Z_{f}^{2}}\right|$$

Here, ${\dot{E}}_{M}$ and ${\dot{E}}_{N}$ represent the electromotive forces of the dual-terminal power supplies, $Z_{M}$ and $Z_{N}$ denote the equivalent impedances from the fault location to the M and N ends, respectively, and $Z_{NO}$ represents the equivalent impedance of the adjacent line at the N terminal.

Assuming ${\dot{E}}_{M}={\dot{E}}_{N}e^{j\delta }$ and denoting δ as the power angle, the ratio of $I_{d}$ to $I_{r}$ is calculated as follows:

(14) $$K=\frac{I_{d}}{I_{r}}=\left|\frac{(Z_{N}+Z_{NO})\cdot {\dot{E}}_{N}e^{j\delta }+Z_{M}\cdot {\dot{E}}_{N}}{(Z_{M}+Z_{NO}+2Z_{f})\cdot {\dot{E}}_{N}e^{j\delta }-(Z_{M}+2Z_{f})\cdot {\dot{E}}_{N}}\right|$$

As indicated in Equation (14), when the line is lightly loaded resulting in δ→0, the condition $K\gg K_{br}$ holds, ensuring that the differential protection maintains high sensitivity. Conversely, when the transmission line is heavily loaded, leading to a large value of δ, and a high-resistance ground fault occurs (i.e., $Z_{f}^{50}\gg Z_{M}+Z_{N}+Z_{NO}$), the condition $K<K_{br}$ is likely to be satisfied. In this scenario, the differential protection may suffer from insufficient sensitivity.

Starting from the output of the DC reactor, the converter valve is represented equivalently as shown within the dashed box in Figure 9. The expression for $Z_{f}^{50}$ is given as follows:

(15) $$Z_{f}^{50}=R_{f}+\frac{\left(Z_{I}+Z_{\mathrm{IP}}^{50})\cdot (2Z_{R}+Z_{I}+Z_{\mathrm{RP}}^{50}+Z_{\mathrm{RN}}^{50}+Z_{\mathrm{IN}}^{50}\right)}{2Z_{R}+2Z_{I}+Z_{\mathrm{IP}}^{50}+Z_{\mathrm{RP}}^{50}+Z_{\mathrm{RN}}^{50}+Z_{\mathrm{IN}}^{50}}$$

Here, $R_{f}$ represents the transition resistance between the two faulted conductors; $Z_{R}$ and $Z_{I}$ denote the equivalent line impedances from the fault point to the converters on both sides, respectively; and $Z_{\mathrm{RP}}^{50}$, $Z_{\mathrm{RN}}^{50}$, $Z_{\mathrm{IP}}^{50}$, and $Z_{\mathrm{IN}}^{50}$ represent the power-frequency equivalent impedances of the four converter valves, respectively.

The equivalent impedance of the converter valve primarily comprises three components, namely the reactor impedance, the converter transformer impedance, and the AC-side system impedance. Their expressions are provided as follows:

(16) $$Z_{\mathrm{eq}1}=\frac{3}{\pi }\omega L_{T}+(2-j\frac{3\mu }{2\pi })\omega L_{T}+\omega L_{d}$$

(17) $$Z_{\mathrm{eq}2}=\frac{3}{4}\sum_{n=6k+1}^{\infty }A_{n}\cdot B_{n}(Z_{n+1}+Z_{n-1})$$

Here, $Z_{\mathrm{eq}1}$ represents the sum of the impedances of the reactor and the converter transformer; $L_{T}$ and $L_{d}$ denote the leakage reactance of the converter transformer and the equivalent inductance of the DC reactor, respectively; μ is the commutation overlap angle; $Z_{\mathrm{eq}2}$ represents the equivalent impedance of the AC-side system; $Z_{n+1}$ and $Z_{n-1}$ denote the *n* + 1 and *n* – 1th harmonic impedances on the AC side of the DC system, respectively; $A_{n}$ and $B_{n}$ are the switching function coefficients for current and voltage, respectively, and their expressions are [25]:

(18) $$A_{n}=\frac{4}{\pi }\frac{1}{n}\sin n(\frac{\pi }{3}-k\mu )$$

(19) $$\left\{\begin{array}{l} B_{n}=\sum_{n=1}^{\infty }A_{n}\cos n\omega t\frac{4}{\pi },\;\;n=6k\pm 1\\ B_{n}=0,\;\;n\neq 6k\pm 1 \end{array}\right.$$

**Figure 11:**
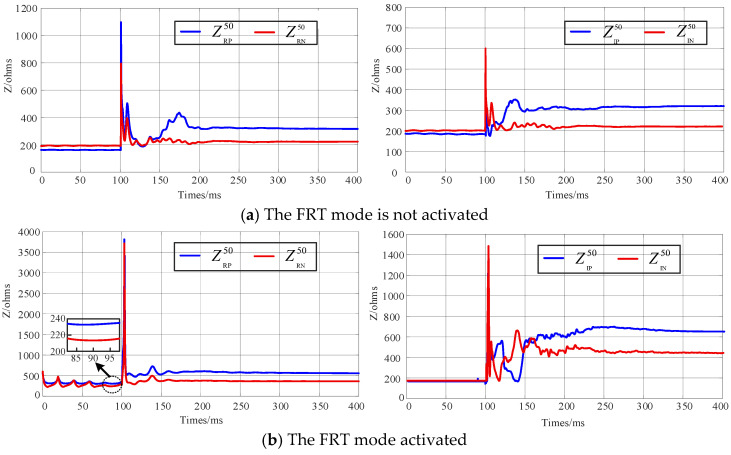
Power-frequency impedance of the valve under different AC-DC ISFs.

As observed from Equations (16)–(19), $Z_{\mathrm{eq}1}$ is predominantly inductive and remains constant at power frequency. The impedance $Z_{\mathrm{eq}2}$ is a complex quantity whose calculation is highly complex and exhibits significant fluctuations. Its value is primarily influenced by the switching states of the converter valves and the degree of electromagnetic coupling between the lines. Literature [26] indicates that after a DC system fault, when operating in the FRT mode, the valve conduction duration is significantly shortened compared to the pre-fault condition. Consequently, it is theoretically expected that equivalent impedance of the converter valve would increase during the steady-state post-fault period. Figure 11 presents the calculated equivalent power-frequency impedance of the valve under different AC-DC ISFs.

As can be seen from Figure 11, the power-frequency equivalent impedance of the converter valve fluctuates within a wide range during the initial fault stage, particularly when the FRT is activated. After the fault reaches a steady state, the impedance stabilizes to a fixed value on the order of hundreds of ohms. Compared to cases where FRT is not activated, the steady-state power-frequency impedance of the valve is higher when the FRT is employed. Consequently, in AC-DC ISF scenarios, AC differential protection is prone to insufficient sensitivity.

### 3.3. Performance of AC Backup Protection Under AC-DC ISF

**Figure 12:**
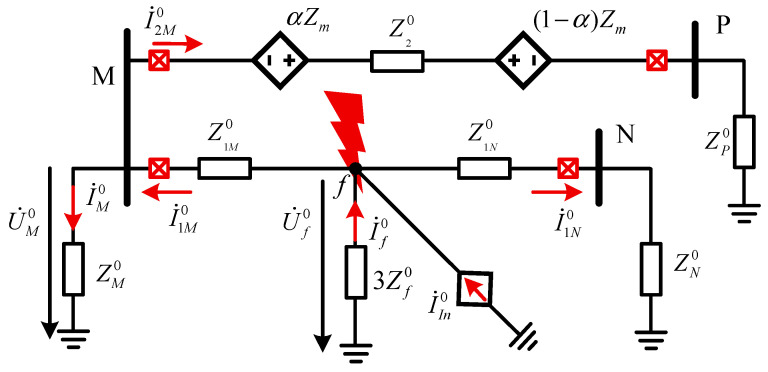
The zero-sequence equivalent network of AC-DC ISF.

HVAC transmission lines must be equipped with comprehensive backup protection for ground faults to respond to faults when primary protection fails. Ground distance protection and zero-sequence current protection are typically employed together as backup protection for ground faults. Among these, ground distance protection has a limited ability to withstand transition resistance and can only reliably clear low-resistance ground faults, while zero-sequence current protection serves as a supplement to clear high-resistance ground faults. The zero-sequence equivalent network for an AC-DC line contact fault is shown in Figure 12. The influence of parallel AC lines on the same tower on the zero-sequence current of the faulted line must be taken into account, as it is significant in multi-circuit transmission systems.

Taking the M-side as an example, the expression for the zero-sequence current ${\dot{I}}_{1M}^{0}$ perceived at the protection location is as follows:

(20) $${\dot{I}}_{1M}^{0}={\dot{I}}_{M}^{0}+{\dot{I}}_{2M}^{0}$$

(21) $${\dot{I}}_{1M}^{0}={\dot{U}}_{M}^{0}\cdot \frac{Z_{2}^{0}+Z_{P}^{0}+Z_{M}^{0}}{Z_{M}^{0}\cdot (Z_{2}^{0}+Z_{P}^{0})}+\frac{({\dot{U}}_{f}^{0}/3Z_{f}^{0}+{\dot{I}}_{In}^{0})\left[\alpha Z_{m}\cdot Z_{1N}^{0}+(1-\alpha )Z_{m}\cdot Z_{1M}^{0}\right]}{\left(Z_{2}^{0}+Z_{P}^{0})\cdot (Z_{1M}^{0}+Z_{1N}^{0}\right)}$$

where $Z_{1M}^{0}$ and $Z_{1N}^{0}$ represent the line zero-sequence impedances on both sides of the fault point, respectively; $3Z_{f}^{0}$ denotes the zero-sequence impedance of the grounding branch; ${\dot{U}}_{f}^{0}$ is the zero-sequence voltage at the AC fault point; ${\dot{I}}_{In}^{0}$ represents the zero-sequence current injected into the AC fault point by the DC system; $Z_{2}^{0}$ signifies the zero-sequence impedance of the parallel AC line on the same tower; and α indicates the proportional distance from the fault point to the M-terminal relative to the total length of the parallel AC line section.

**Figure 13:**
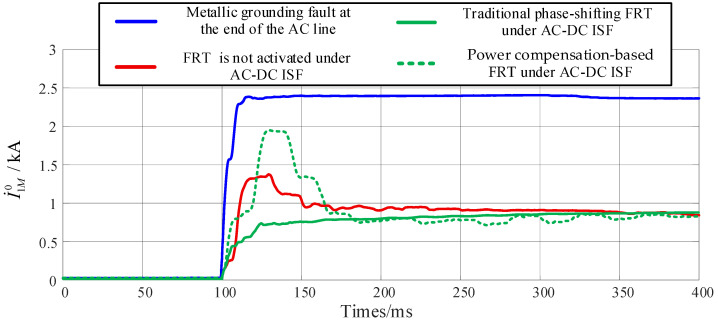
The RMS of zero-sequence current under different fault scenarios.

Compared to single-circuit transmission lines, the additional term ${\dot{I}}_{2M}^{0}$ in multi-circuit transmission lines is proportional to α. Specifically, when the fault occurs at the terminal of the parallel AC line section, the value of ${\dot{I}}_{2M}^{0}$ reaches its maximum. Consequently, the setting of zero-sequence current protection must be re-coordinated based on the length of the parallel AC line section. Parameter ${\dot{I}}_{In}^{0}$ can be adjusted by the DC control system, thereby influencing the performance of AC zero-sequence protection. The RMS of zero-sequence current serves as the characteristic quantity for zero-sequence current protection. Figure 13 illustrates the RMS values of zero-sequence current under different fault scenarios, where the AC-DC ISF is configured as a metallic fault at the head of line. The RMS calculation for zero-sequence current employs a sliding window design, utilizing a fixed-length window of one cycle. This window begins sliding from the sampling point coinciding with the fault inception. As each new sampling point arrives, the window discards the oldest point and incorporates the newest one, thereby enabling real-time RMS computation.

Zero-sequence current protection is typically set with reference to a metallic grounding fault at the end of the AC line for its Zone I setting. The simulation results in Figure 13 demonstrate that even when a fault occurs at the head of line (where protection sensitivity is highest), the measured zero-sequence current RMS values perceived by the AC protection are significantly lower than the Zone I threshold, regardless of whether no FRT, conventional phase-shifting FRT, or power compensation-based FRT is employed. Under such conditions, the fault can only be cleared by backup protection with a long-time delay. Furthermore, when fault distance and transition resistance are considered, the sensitivity and rapid-response performance of the zero-sequence current protection will be further degraded.

## 4. Protection-Control Cooperation FRT Strategy for AC-DC ISF

### 4.1. Implementation Method

In the AC-DC ISF scenario, both traditional longitudinal current differential protection and Zone I zero-sequence current protection suffer from insufficient sensitivity. Relying on backup protection with poor speed to clear faults may lead to the activation of DC converter valve protection and consequently cause its shutdown. It is noteworthy that, in the AC-DC ISF, the zero-sequence current detected by the protection system contains a zero-sequence component injected by the DC system. This component ${\dot{I}}_{In}^{0}$ can be adjusted by modifying the control strategies of the DC system. Without altering the existing configuration of AC line protection, the flexible regulation capability of the DC system can be fully utilized to modify the injected ${\dot{I}}_{In}^{0}$, thereby achieving proactive activation of the Zone I zero-sequence current protection.

**Figure 14:**
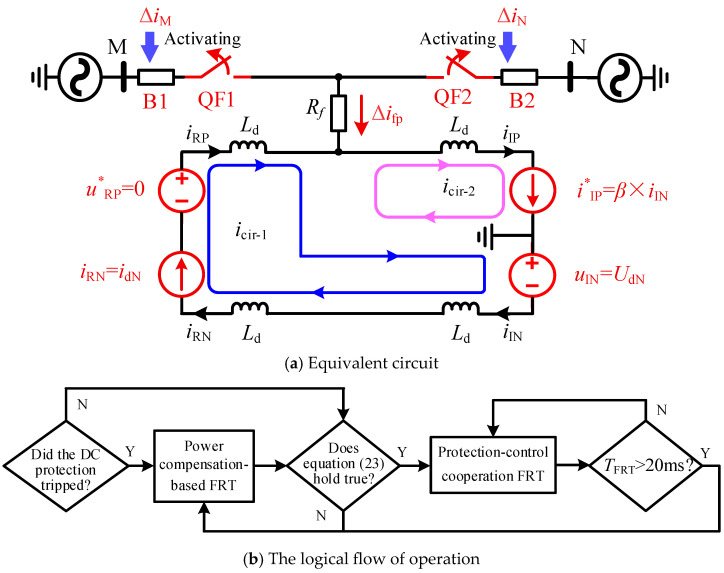
Fault equivalent circuit and operating logic of the protection-control cooperation FRT.

Valves RP, RN, and IN maintain the control mode from Figure 6 to ensure power transmission of the non-faulted pole. The current reference value of valve-IP is set to $\beta \times i_{\mathrm{IN}}$. This strategy is referred to as the protection-control cooperation FRT strategy, and its equivalent circuit is shown in Figure 14a, where $\Delta i_{\mathrm{fp}}=(\beta -1)\times i_{\mathrm{dN}}$. In the single-phase-to-pole ISF scenario, ${\dot{I}}_{In}^{0}=\Delta i_{\mathrm{fp}}=(\beta -1)\times i_{\mathrm{dN}}$, and increasing the β can enhance the sensitivity of zero-sequence protection. However, considering the avoidance of prolonged overcurrent conditions in valve-IP, the β should not be excessively large and can be described within the range as follows:

(22) $$C_{1}/i_{\mathrm{dN}}+1\leq \beta \leq C_{2}$$

where $C_{1}$ denotes the current value that ensures the tripping of zero-sequence protection when the AC-DC ISF occurs at the end of the same tower line, while $C_{2}$ represents the maximum transient overcurrent that the converter can withstand.

The protection-control cooperation strategy requires the support of AC-DC ISF detection criteria to guide the activation and deactivation of the strategy. This paper employs a technologically mature criterion based on the detection of AC components in DC line:

(23) $$U_{\mathrm{DC}}^{\mathrm{FF}}>K_{re}\times U_{\mathrm{RE}}^{\mathrm{FF}}$$

Here, $U_{\mathrm{DC}}^{\mathrm{FF}}$ represents the AC power-frequency component detected in the DC line, $U_{\mathrm{RE}}^{\mathrm{FF}}$ denotes the setting reference value, and $K_{re}$ corresponds to the reliability coefficient. Drawing on the established practice for power system overcurrent protection, this study employs a typical value of 1.2.

According to literature [27], when a single-pole metallic grounding fault occurs on an AC line parallel to the DC line on the same tower, the maximum AC power-frequency component will be coupled into the DC line; therefore, the value of $U_{\mathrm{RE}}^{\mathrm{FF}}$ must be determined with reference to this scenario. To prevent the I-side converter from sustaining prolonged overcurrent damage, the protection-control cooperation FRT strategy is activated for only 20 ms after criterion (23) is satisfied. The proposed strategy can be coordinated with power compensation-based FRT strategies to cover all fault types in hybrid AC/DC transmission systems. Its activation logic is illustrated in Figure 14b.

### 4.2. Simulation Verification

**Figure 15:**
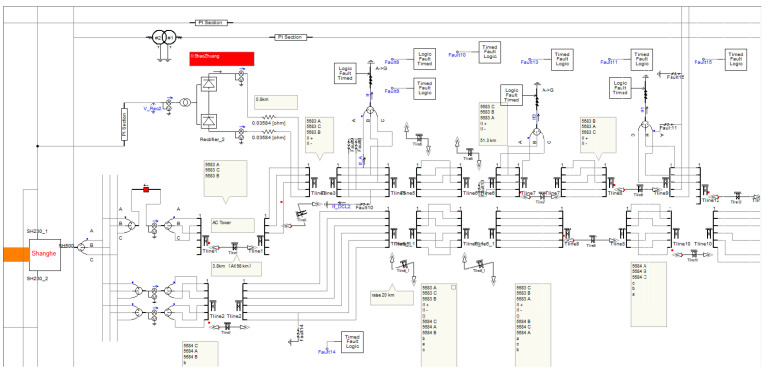
The screenshot of the hybrid AC/DC transmission system model based on PSCAD.

In AC-DC ISF scenarios, the performance of DC primary protection is influenced by the fault initial phase angle of the AC line, which may lead to a failure to operate. Figure 15 displays the screenshot of the hybrid AC/DC transmission system model developed by the authors, implemented in PSCAD version 4.5. The procedure is as follows: first, the fault generation module in the model simulates real-world power system fault scenarios; then, a decision module, as defined by (23), determines whether to adjust the DC controller within the model; finally, the fault current calculation module is utilized to observe the transient zero-sequence current in the AC line, thereby validating the impact of active control on the AC zero-sequence protection.

**Figure 16:**
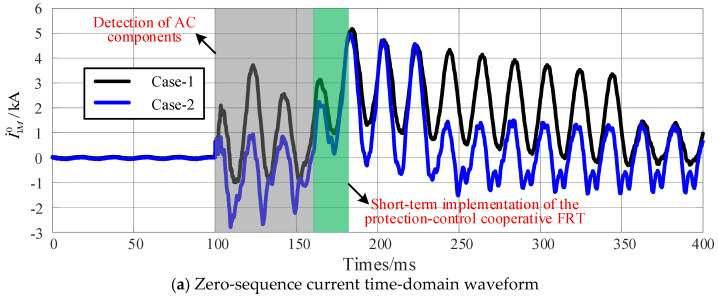

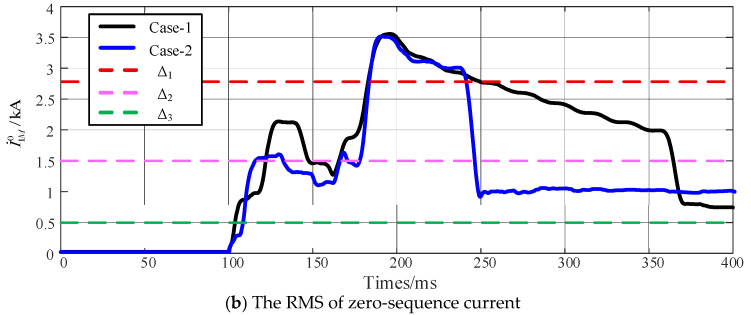
Zero-sequence current during protection-control cooperation FRT strategy.

The scenarios where DC primary protection trips and does not trip are referred to as Case-1 and Case-2, respectively. A single-pole-to-single-phase metallic fault is applied at the end of the hybrid AC-DC same-tower section, during which the transient zero-sequence current is depicted in Figure 16.

Regardless of whether the DC primary protection trips, the AC voltage component can be detected in the DC line under AC-DC ISF. The protection- control cooperation FRT strategy is activated 60 ms after fault, resulting in an increased amplitude of the zero-sequence current as illustrated in Figure 16a. In Figure 16b, $\Delta_{1}$, $\Delta_{2}$, and $\Delta_{3}$ represent the threshold values for Zone I, Zone II, and Zone III of the zero-sequence current protection, respectively. After the initiation of the protection-control cooperation strategy, the RMS value of the zero-sequence current transiently exceeds threshold A within milliseconds of the regulation process, thereby ensuring the immediate tripping of the AC circuit breaker by the zero-sequence protection section-I.

## 5. Conclusions

The conventional phase-shift strategy causes a complete interruption of power transmission in SM-LCC-HVDC systems during single-pole fault clearance, resulting in significant impact on the AC system. To address single-pole fault clearance in such systems, this paper proposes an improved power compensation-based FRT strategy. By modifying the control strategy of the faulted-pole converter to limit fault current, while maintaining the original control strategy of the non-faulted pole to sustain power transmission, the DC system can transmit half of the rated active power during the FRT period, thereby substantially reducing reactive power redundancy. Furthermore, a protection-control cooperation FRT strategy is introduced, which activates during AC-DC ISFs by injecting an additional zero-sequence component into the AC system. During the FRT process, the Zone I zero-sequence current protection is proactively triggered, enabling rapid tripping of the AC circuit breaker. It should be emphasized that the implementation of both fault detection and control strategies within the system relies fundamentally on sensors. The strategy proposed in this paper constitutes a fundamental theoretical framework, which is extensible for industrial applications and can provide guidance for the development of novel sensors in power systems.

## Figures and Tables

**Table 1:** System parameters.

AC System	DC System A	DC System B
*U*_N_ = 500 kV (50 Hz)	*U*_NA_ = ±200 kV	*U*_NB_ = ±200 kV
Z_Th(1)_ = 2.03 Ω	*I*_DC_ = 2.5 kA	*I*_DC_ = 1.9 kA
*X*/*R* = 9.23	*L*_d_ = 150 mH	*L*_d_ = 150 mH
Solidly grounded	*P*_DC_ = 500 MW	*P*_DC_ = 380 MW
Length = 98 km	Length = 201 km	Length = 142 km

## Data Availability

Data available in a publicly accessible repository.
